# Transcriptomic and metabolomic analysis of peri-tumoral hepatic tissue in hepatocellular carcinoma: unveiling the molecular landscape of immune checkpoint therapy resistance

**DOI:** 10.3389/fphar.2023.1304996

**Published:** 2024-01-03

**Authors:** Huaqiang Bi, Kai Feng, Xiaofei Wang, Ping Zheng, Chengming Qu, Kuansheng Ma

**Affiliations:** Institute of Hepatobiliary Surgery, Southwest Hospital, Third Military Medical University, Chongqing, China

**Keywords:** hepatocellular carcinoma, immune checkpoint therapy, Atezolizumab, COMMD3-BMI1, Dephospho-CoA, therapy resistance, transcriptomics, metabolomics

## Abstract

**Background:** Hepatocellular carcinoma (HCC) often resists traditional treatments, necessitating new therapeutic approaches. With immune checkpoint therapy emerging as a promising alternative, understanding its resistance mechanisms becomes crucial.

**Methods:** Using 22 samples from 11 HCC patients, we conducted a comprehensive transcriptomic and metabolomic analysis of peri-tumoral hepatic tissues from those treated with Atezolizumab.

**Results:** We identified significant metabolic alterations and a correlation between the COMMD3-BMI1 gene and Dephospho-CoA metabolite. Findings suggest these as potential markers for therapeutic resistance, as evidenced by upregulated COMMD3-BMI1 and downregulated Dephospho-CoA in non-responsive patients, with animal models further supporting these observations.

**Discussion:** The study highlights COMMD3-BMI1 and Dephospho-CoA as critical actors in immune checkpoint therapy resistance in HCC, providing insights and potential pathways for more effective therapeutic strategies.

## Introduction

Hepatocellular carcinoma (HCC) ranks as the third leading cause of cancer-related deaths globally, with an alarming incidence rate of approximately 905,677 new cases and 830,180 deaths in 2020 alone (Gao et al., 2023). The incidence is particularly pronounced in Eastern Asia and sub-Saharan Africa due to the high prevalence of chronic hepatitis B and C infections, which contribute to approximately 80% of HCC cases (Nordenstedt et al., 2010). The molecular mechanism underlying HCC is intricate, involving a cascade of genetic and epigenetic alterations leading to the deregulation of crucial cellular pathways controlling cell proliferation, apoptosis, and DNA repair (Herceg and Vaissière, 2011). Diagnostics of HCC primarily relies on a combination of imaging techniques—like ultrasound, CT, and MRI—and serum biomarkers, chiefly alpha-fetoprotein (AFP). Nevertheless, AFP’s sensitivity ranges from 41% to 65%, and specificity from 80% to 94%, thereby limiting its diagnostic efficacy, especially in the early stages of the disease (Galle et al., 2019). Treatment modalities are diverse, ranging from surgery, radiotherapy, and chemotherapy to targeted therapies and immunotherapy. Surgical methods, including resection and transplantation, are often constrained by the tumor’s size and stage, patients’ liver function, and overall health status. The 5-year survival rate for localized HCC is approximately 31%, which plummets to a meager 2% for distant metastasis (Jariwala and Sarkar, 2016).

In recent years, immunotherapy has burgeoned as a promising alternative, with agents like Atezolizumab heralding significant clinical benefits. Immune checkpoint inhibitors (ICIs) function by revitalizing the host’s immune response against tumor cells. Agents like Atezolizumab have been at the forefront, heralding significant clinical benefits by meticulously targeting and inhibiting the PD-L1 checkpoint receptor. The mechanism encompasses reinvigorating the immune system, thereby facilitating the identification and subsequent destruction of cancer cells, which usually adeptly camouflage themselves from the body’s immune surveillance (Rizzo et al., 2021). Atezolizumab, a fully humanized, engineered monoclonal antibody of IgG1 isotype, is specifically designed to bind to PD-L1 and block its interactions with both PD-1 and B7.1 receptors (Cheng et al., 2019). This interference restores anti-cancer immune responses by enabling the activation of T-cells and the influx of activated T-effector cells into the tumor microenvironment, thereby promoting the death of tumor cells. Clinical trials have evidenced that Atezolizumab improves the overall survival rates and exhibits a favorable safety profile in a subset of HCC patients.

However, immunotherapy, while groundbreaking, is not devoid of challenges. One of the prominent hurdles is the heterogeneity in response rates among patients. Statistics reveal that a significant proportion of patients—approximately 70%–85%—do not respond effectively to immune checkpoint inhibitors, including Atezolizumab (Ganesh et al., 2019). This non-responsiveness could be attributed to various factors including genetic mutations, expression levels of PD-L1, and the overall tumor microenvironment. Moreover, resistance to immune checkpoint therapy, both inherent and acquired post-treatment, poses a substantial impediment to the success of immunotherapy in HCC. The mechanisms underpinning this resistance are complex and multifaceted, encompassing alterations in antigen presentation, defects in the interferon signaling pathway, and the expression of alternative immune checkpoints (Hack et al., 2020; Shukla et al., 2021). Understanding these mechanisms is paramount as it provides a foundation for developing strategies to overcome resistance, thus enhancing the efficacy of immune checkpoint therapy in HCC.

Notably, while prior research endeavors have provided invaluable insights into the tumor tissues themselves, the peri-tumoral hepatic tissue—a pivotal yet often overlooked component—has not been meticulously explored (Tang et al., 2016). Given its crucial role and dynamic nature, understanding the molecular and cellular alterations within the peri-tumoral hepatic tissue is imperative. Our study aims to shed light on this uncharted territory, offering an in-depth transcriptomic and metabolomic analysis of peri-tumoral hepatic tissue in HCC patients resistant to Atezolizumab, thereby unveiling novel mechanisms of resistance and paving the way for innovative therapeutic strategies and interventions.

## Methods

### Sample collection

Initially, 21 patients diagnosed with hepatocellular carcinoma (HCC) were prospectively enrolled in the study over a 12-month period. Out of these, 11 patients, providing a total of 22 samples, were selected for further analysis. Moreover, the study protocol was reviewed and approved by the Institutional Review Board (IRB) of our hospital, ensuring adherence to ethical guidelines and standards. Patient inclusion was meticulously adhered to specific criteria: ages between 18 and 75, a histopathological confirmed diagnosis of HCC, no previous exposure to immune checkpoint inhibitors or related immunotherapy, adequate organ function demonstrated through a comprehensive metabolic panel, and an expected survival timeframe extending beyond 12 weeks. Additionally, our experiment received ethical approval from our hospital’s review board. Concurrently, exclusion parameters were set to omit pregnant or breastfeeding women, individuals with autoimmune diseases or immunodeficiency, and those with malignancies other than HCC. Upon the application of these stringent inclusion and exclusion parameters, the initial cohort was refined down to 12 patients who met the criteria robustly. From these selected participants, peri-tumoral hepatic tissue samples were diligently collected both prior to the administration of Atezolizumab and following the manifestation of resistance to the therapy. The collection timeline was diligently designed to allow for a nuanced understanding of the molecular shifts occurring in response to the treatment and subsequent resistance development. For the preservation of the integrity of the collected tissue samples, each specimen was immediately submerged in liquid nitrogen upon extraction. This rapid-freezing process was crucial for preventing the degradation of RNA, proteins, and other vital cellular components, thereby ensuring that the samples would be viable for the subsequent transcriptomic and metabolomic analyses planned for the study. Each frozen sample was then carefully transferred and stored in a −80°C freezer until the commencement of the analysis phase.

### Evaluation of immunotherapy response

The response to immunotherapy was meticulously assessed based on established clinical criteria to discern between responders (Response) and non-responders (Non-Response) to the Atezolizumab treatment.

Patients were categorized as responders if they exhibited a partial or complete response to the treatment, as delineated by the Response Evaluation Criteria in Solid Tumors (RECIST) version 1.1 (Eisenhauer et al., 2009). Specifically:

Complete Response (CR): Total disappearance of all target lesions, with no new lesions identified. No evidence of non-target lesion progression is noted, and tumor marker levels are within the normal range. Partial Response (PR): At least a 30% decrease in the sum of diameters of target lesions, taking as reference the baseline sum diameters, with no evidence of progression in non-target lesions or the emergence of new lesions. For Non-Response Criteria, patients were identified as non-responders in cases of progressive disease or stable disease as follows: Progressive Disease (PD): A minimum 20% increase in the sum of diameters of target lesions, with an absolute increase of at least 5 mm, or the appearance of one or more new lesions. Alternatively, progression in non-target lesions also constitutes PD. Stable Disease (SD): Neither sufficient shrinkage to qualify for PR nor sufficient increase to qualify for PD, taking as reference the smallest sum diameters while on the study.

### RNA extraction and RNA-seq sequencing

Upon the commencement of sample analysis, RNA extraction from the meticulously collected peri-tumoral hepatic tissues initiated, deploying the TRIzol Reagent method due to its efficacy in yielding high-quality RNA. Each frozen tissue sample was homogenized in TRIzol, and RNA was subsequently isolated following a series of centrifugation steps that segregated RNA from DNA and proteins, thus ensuring the acquisition of pure RNA. For RNA-seq sequencing, the extracted RNA underwent a quality check using the Agilent 2100 Bioanalyzer to ascertain the integrity and concentration of RNA. Following verification, libraries were prepared using the Illumina TruSeq RNA Sample Preparation Kit, adhering strictly to the manufacturer’s protocol. The prepared libraries were then sequenced on the Illumina HiSeq 2000 platform, which facilitated the generation of paired end reads, providing comprehensive coverage and depth for accurate transcriptome profiling.

#### RNA-seq quantification

For the RNA-seq data quantification, the study employed the nf-core/rnaseq pipeline, a highly efficient and reproducible tool designed for the analysis and quantification of high-throughput RNA-sequencing data (Ewels et al., 2020). This sophisticated pipeline is open-source and supports the latest tools and formats which facilitate a flexible and reproducible analysis of the RNA-seq data. Upon receiving the raw sequencing data, the initial step involved quality control checks using FastQC to ensure the integrity and quality of the raw reads (Brown et al., 2017). The nf-core/rnaseq pipeline was then configured to align the reads to the reference genome using the STAR aligner due to its high accuracy and efficiency in mapping reads to a reference genome. The aligned reads were then quantified at the gene level using the featureCounts function incorporated within the pipeline (Dobin et al., 2013). featureCounts is a highly efficient general-purpose read summarization program that counts mapped reads for genomic features such as genes, exons, promoter, gene bodies, genomic bins, and chromosomal locations. Following the quantification, the RNA-seq count data underwent normalization to adjust for sequencing depth and RNA composition. Normalization is crucial for removing biases that could affect the comparison between samples. After normalization, differential expression analysis was conducted to identify genes that were expressed differently between sample groups. The DESeq2 package was utilized for this purpose due to its robustness in analyzing count data and identifying differentially expressed genes (Love et al., 2014).

### Metabolomic analysis

The process initiated with the meticulous homogenization of the hepatic tissue samples, employing a 1:3 (v/v) cold methanol-water mixture from Sigma-Aldrich (St. Louis, MO, USA). This mixture efficaciously facilitated the extraction of a wide array of metabolites. Following this, a chloroform (Fisher Scientific, Hampton, NH, USA) and water phase separation technique was applied, effectively segregating hydrophilic and lipophilic metabolites. Post-centrifugation, both aqueous (containing hydrophilic metabolites) and organic (harboring lipophilic metabolites) layers were isolated and carefully collected. After the extraction, the acquired layers were evaporated under nitrogen conditions using a gentle nitrogen evaporator (Organomation, Berlin, MA, USA). The residues were then reconstituted meticulously; acetonitrile-water (ACN-H2O) mixture from Honeywell (Charlotte, NC, USA) was used for hydrophilic metabolites, while a combination of isopropanol-acetonitrile (IPA-ACN) from Thermo Fisher Scientific (Waltham, MA, USA) was employed for lipophilic ones. The liquid chromatography-mass spectrometry (LC-MS) analysis engaged an Acquity UPLC system (Waters Corporation, Milford, MA, USA) paired with a Synapt G2-Si HDMS mass spectrometer (Waters Corporation, Milford, MA, USA). Hydrophilic metabolites were channeled through a BEH Amide column (Waters Corporation, Milford, MA, USA) with a gradient mixture of water and acetonitrile, each containing 0.1% formic acid from Sigma-Aldrich (St. Louis, MO, USA). Lipophilic metabolites utilized a BEH C8 column (Waters Corporation, Milford, MA, USA) with a gradient of acetonitrile and isopropanol, both containing 0.1% formic acid. The mass spectrometer operated in both positive and negative ion modes to ensure a comprehensive detection of metabolites. The subsequent data processing, including peak detection and alignment, utilized the Progenesis QI software (Nonlinear Dynamics, Newcastle upon Tyne, UK). Identified metabolites were annotated, verified against the Human Metabolome Database (HMDB) and METLIN, ensuring a thorough and accurate metabolomic profile for each sample in the study.

### Experimental animals and hepatocellular carcinoma model

FVB mice were acquired from the Institute of Zoology, Chinese Academy of Sciences (Beijing, China), and were housed under specific pathogen-free conditions, with free access to food and water. All animal experiments were conducted in accordance with the guidelines approved by the Animal Ethics Committee of our institution. After a standardized acclimatization period, hepatocellular carcinoma (HCC) induction commenced. To induce HCC, mice were subjected to a carefully calibrated dose regimen of diethylnitrosamine (DEN, Sigma-Aldrich, St. Louis, MO, USA), a potent hepatocarcinogen. DEN was administered through intraperitoneal injection starting with a dose of 20 mg/kg body weight when the mice were 15 days old, followed by a dose of 30 mg/kg in the third week, and then 50 mg/kg for the last 6 weeks. For the administration of immunotherapy, the anti-PD-L1 monoclonal antibody Clone 10F.9G2, Bio X Cell, West Lebanon, NH, USA was selected, with intraperitoneal injections of 100 µg per mouse administered twice a week.

### RT-PCR analysis

Complementary DNA (cDNA) synthesis was performed with 1 µg of total RNA using the High-Capacity cDNA Reverse Transcription Kit (Applied Biosystems, Foster City, CA, USA). For the PCR amplification, specific primers designed for the BMI1 gene were utilized. The forward primer sequence was 5′-ACT​ACA​CGC​TAA​TGG​ACA​TTG​CC-3′, and the reverse primer sequence was 5′-CTC​TCC​AGC​ATT​CGT​CAG​TCC​A-3′. The PCR conditions were set with an initial denaturation step at 95°C for 3 min, followed by 40 cycles of denaturation at 95°C for 30 s, annealing at 60°C for 30 s, and extension at 72°C for 30 s, with a final extension step at 72°C for 5 min. The relative expression levels of COMMD3-BMI1 were quantified using the 2^−ΔΔCT^ method, normalized to the expression of the housekeeping gene GAPDH, the forward primer sequence was 5′-CAT​CAC​TGC​CAC​CCA​GAA​GAC​TG-3′, and the reverse primer sequence was 5′-ATG​CCA​GTG​AGC​TTC​CCG​TTC​AG-3′.

### Statistical analysis

A comprehensive statistical analysis was meticulously conducted to discern significant differences and patterns within the accumulated data. The Python3.7 programming language, renowned for its versatility and the extensive library support for data analysis and statistics, was deployed for this crucial phase of the study. PCA was carried out using the HiPlot visualization tool, a robust Python library designed for high-dimensional data (Li et al., 2022). PCA facilitated the reduction of dimensionality of our dataset while retaining the variance in the data. This approach allowed for the identification and visualization of patterns and clusters within the data, thereby providing an initial understanding of the underlying structure and relationships within the observed variables. HiPlot was selected for its interactive visualization features, enabling more efficient exploration and interpretation of PCA results. The paired T-test was chosen for its appropriateness in analyzing the means of two related groups. The assumption of normality was tested and confirmed, and subsequently, the T-test was applied to evaluate whether the mean difference between paired observations was statistically significant. Variable Importance in Projection (VIP) Scores were calculated to identify significant variables contributing to the variation and classification in the PCA model. The VIP value for each variable was computed as a weighted sum of the squared correlations between the variable and the principal components. A variable with a VIP scores greater than 1.0 was considered important for the projection. This calculation facilitated the prioritization of significant metabolites and genes in the dataset, providing insight into the elements driving the separation and classification observed in the PCA plots.

## Results

### Patient clinical information and metabolomics

**FIGURE 1:**
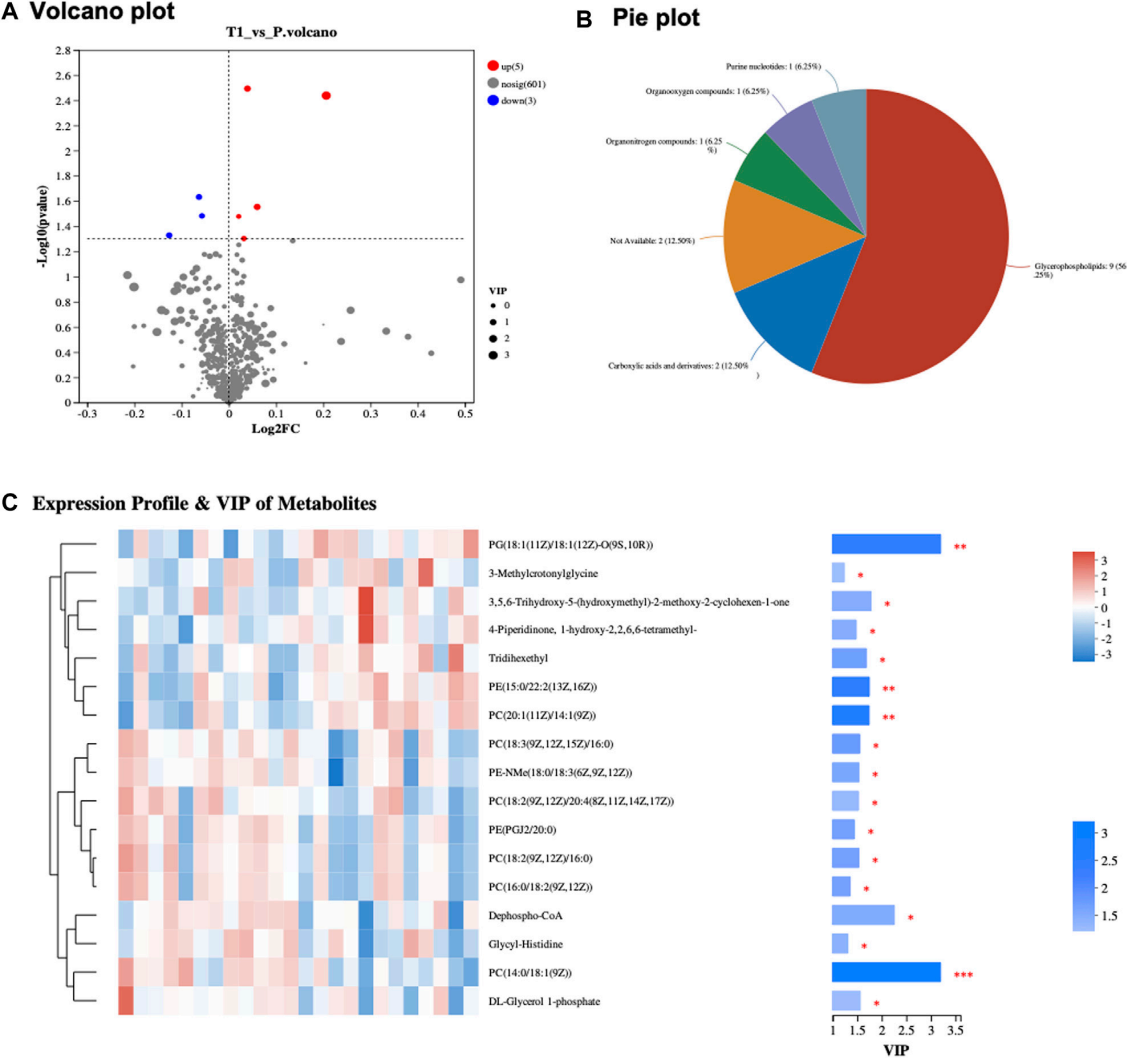
**(A)** Volcano Plot of Metabolites: Displays significant metabolic alterations between pre- and post-treatment samples. Upregulated metabolites are in red, and downregulated ones are in blue, with the x-axis showing fold change and the y-axis depicting −log10 (*p*-value). **(B)** Metabolite Classification Pie Chart: Visual representation of significantly expressed metabolites, segmented into categories. Glycerophospholipids comprise 56.25%, Carboxylic acids and derivatives 12.5%, with Purine nucleotides, Organoxygen compounds, and Organonitrogen compounds each constituting 6.25%. **(C)** Heatmap of Expression Profile and VIP of Metabolites: Depicts expression profiles and VIP scores of metabolites, emphasizing PC (14:0/18:1 (9Z)), PG (18:1 (11Z)/18:1 (12Z)-O (9S,10R)), and Dephospho-CoA.

**FIGURE 2:**
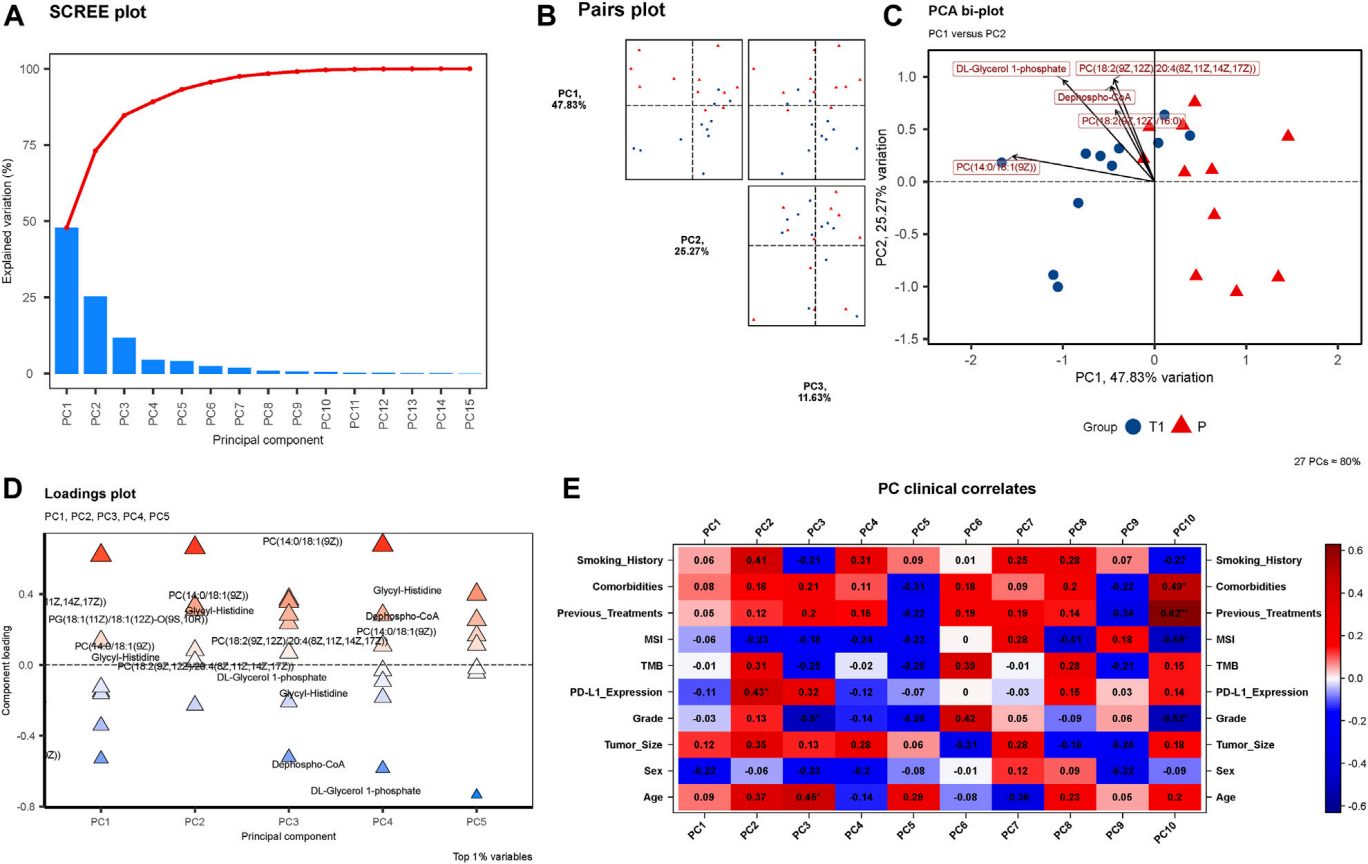
**(A)** SCREE Plot: Displays the explained variation across 15 Principal Components (PCs), showcasing a moderate decline in variation explained by each subsequent PC. **(B)** Pairs Plot: Illustrates the distribution of data points in the space defined by PC1 (47.83%), PC2 (25.27%), and PC3 (11.63%). **(C)** PCA Bi-plot: Highlights four prominent metabolites in the PCA—Dephospho-CoA, PC(14:0/18:1 (9Z)), DL-Glycerol 1-phosphate, and PC(18:2 (9Z,12Z)/20:4 (8Z,11Z,14Z,17Z)). **(D)** Loadings Plot: Presents the component loadings for each metabolite, with the significant four metabolites still notable in contributing to PCs. **(E)** PC Clinical Correlates: Visualizes correlations between 10 PCs and various clinical factors, with only MSI and Sex negatively correlated in PC1, PC2, and PC3.

The study incorporated a cohort comprising 22 distinct samples, originating from 11 patients, with each patient contributing a pair of samples collected before and after Atezolizumab treatment (Supplementary Table S1). This cohort featured a varied patient demographic with ages ranging from 40 to 64 years, involving both genders (six males and five females). Tumor sizes in these patients were diverse, ranging from 1.17 to 9.49 cm, with tumor grades spanning from G2 to G4, indicative of the tumor’s heterogeneity. All patients exhibited non-responsiveness to Atezolizumab treatment, with varying levels of PD-L1 expression, ranging from low to medium, and tumor mutational burden (TMB) ranging from low to high. The microsatellite status within the cohort predominantly showcased microsatellite stability (MSS), with a few instances of high microsatellite instability (MSI-H). Previous treatments the patients underwent before the study were diverse, including chemotherapy, surgery, radiation, or none, and comorbidities like diabetes and hypertension were also recorded, with some patients having a smoking history**.** In the metabolomic assessment of peri-tumoral hepatic tissues collected pre- and post-Atezolizumab treatment, a revealing volcanic plot was elucidated in Figure 1A, visually representing the significant metabolic alterations observed. The plot designated metabolites that were upregulated (depicted in red) and those that were downregulated (illustrated in blue), providing a clear demarcation of the metabolic shifts post-treatment. The subsequent categorization of these significantly altered metabolites, as delineated in Figure 2A, presented a predominant group of Glycerophospholipids accounting for a substantial 56.25% of the changes. Carboxylic acids and their derivatives also held a significant portion, constituting 12.5% of the altered metabolic profile. Meanwhile, other categories such as Purine nucleotides, Organoxygen compounds, and Organonitrogen compounds each comprised 6.25% of the total, collectively contributing to the intricate metabolic landscape observed in the hepatic tissues following treatment. In a further nuanced examination showcased in Figure 1C, the study focused on the expression profile and Variable Importance in Projection (VIP) of metabolites. Here, the heatmap vividly displayed the variance in expression levels, with the three most significant metabolites emerging as PC (14:0/18:1 (9Z)), PG (18:1 (11Z)/18:1 (12Z)-O (9S,10R)), and Dephospho-CoA (Supplementary Table S2).

### Metabolites PCA and KEGG enrichment analysis

In the exploratory PCA of metabolites, Figure 2A unveils a SCREE plot, with the y-axis denoting “Explained Variation” and the x-axis listing 15 Principal Components (PCs). The explained variation descends progressively with each subsequent PC, exhibiting a moderate slope rather than a sharp decline, indicative of the distribution of variance across the PCs. Figure 2B presents a Pairs plot incorporating PC1 (explaining 47.83% of the variance), PC2 (25.27%), and PC3 (11.63%). The plot visually exemplifies the relationships and distribution of data points in the space defined by these principal components, providing insight into the structure and variance within the metabolomic data. Furthermore, the PCA bi-plot illustrated in Figure 2C identifies the four metabolites that are most prominent within the principal component analysis: Dephospho-CoA, PC (14:0/18:1 (9Z)), DL-Glycerol 1-phosphate, and PC(18:2 (9Z,12Z)/20:4 (8Z,11Z,14Z,17Z)). Figure 2D, the Loadings plot, graphically represents the importance of each variable (metabolites) to the principal components, with the y-axis indicating “Principal Component” and the x-axis signifying “Component Loading”. The previously mentioned four metabolites maintain their significance in this representation, further emphasizing their importance in the observed metabolic alterations. Lastly, Figure 2E delineates the PC Clinical Correlates, depicting the relationships between the 10 PCs and various clinical factors including Smoking History, Comorbidities, Previous Treatments, MSI, TMB, PD-L1 Expression, Grade, Tumor Size, Sex, and Age. Notably, within PC1, PC2, and PC3, only MSI and Sex display negative correlations, while the remaining factors exhibit positive correlations.

**FIGURE 3:**
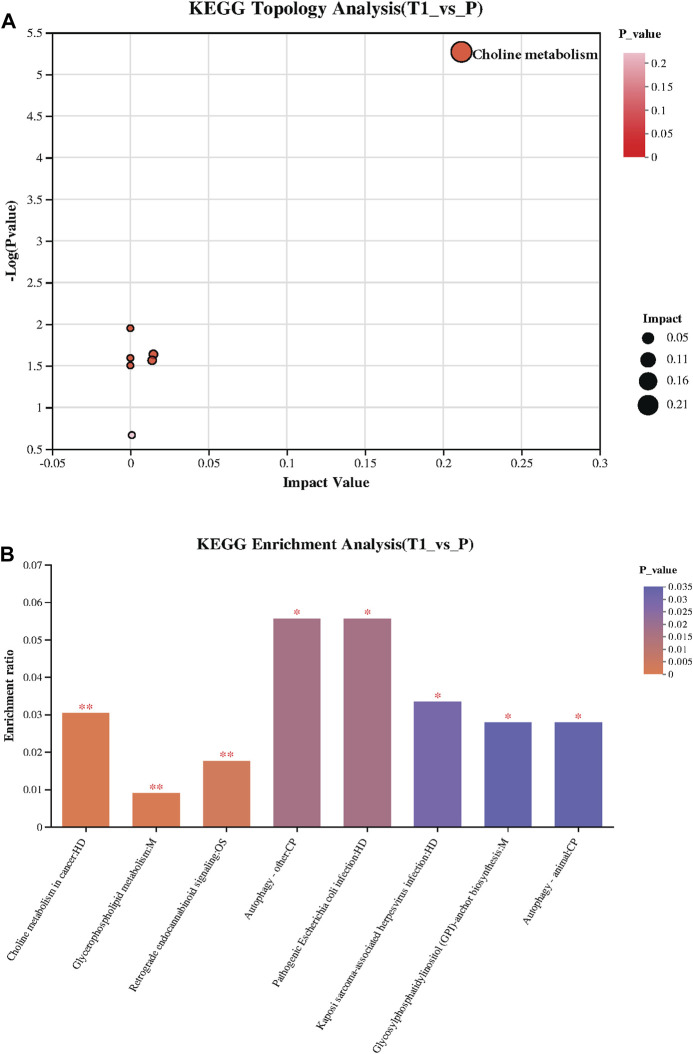
**(A)** KEGG Topology Analysis: Visualizes a comparison between pre-treatment (T1) and post-treatment (P) samples, with the x-axis indicating the Impact Value and the y-axis showing −log (*p* value). A specific category within the Topology analysis is extremely significant, highlighting its potential importance in the observed post-treatment metabolic changes. **(B)** KEGG Enrichment Analysis: Presents the pathways significantly enriched with the identified metabolites, with “Choline metabolism in cancer,” “Glycerophospholipid metabolism,” and “Retrograde endocannabinoid signaling” emerging as the most significant.

The KEGG Topology analysis is depicted in Figure 3A, presenting a comparative perspective between the pre-treatment (T1) and post-treatment (P) samples. On the x-axis, the graph displays the impact value while the y-axis represents −log (*p* value). Remarkably, one specific category within the Topology analysis demonstrates extreme significance, standing out prominently in the visual representation of the data, signaling its potential importance and impact on the metabolic changes observed post-treatment. Following, Figure 3B provides a visual summary of the KEGG enrichment analysis, spotlighting the pathways that are most significantly enriched with the identified metabolites. Notably, the analysis reveals that the most significant pathways enriched are “Choline metabolism in cancer”, “Glycerophospholipid metabolism”, and “Retrograde endocannabinoid signaling”.

### Transcriptomic analysis of peri-tumoral hepatic tissues pre- and post-treatment

**FIGURE 4:**
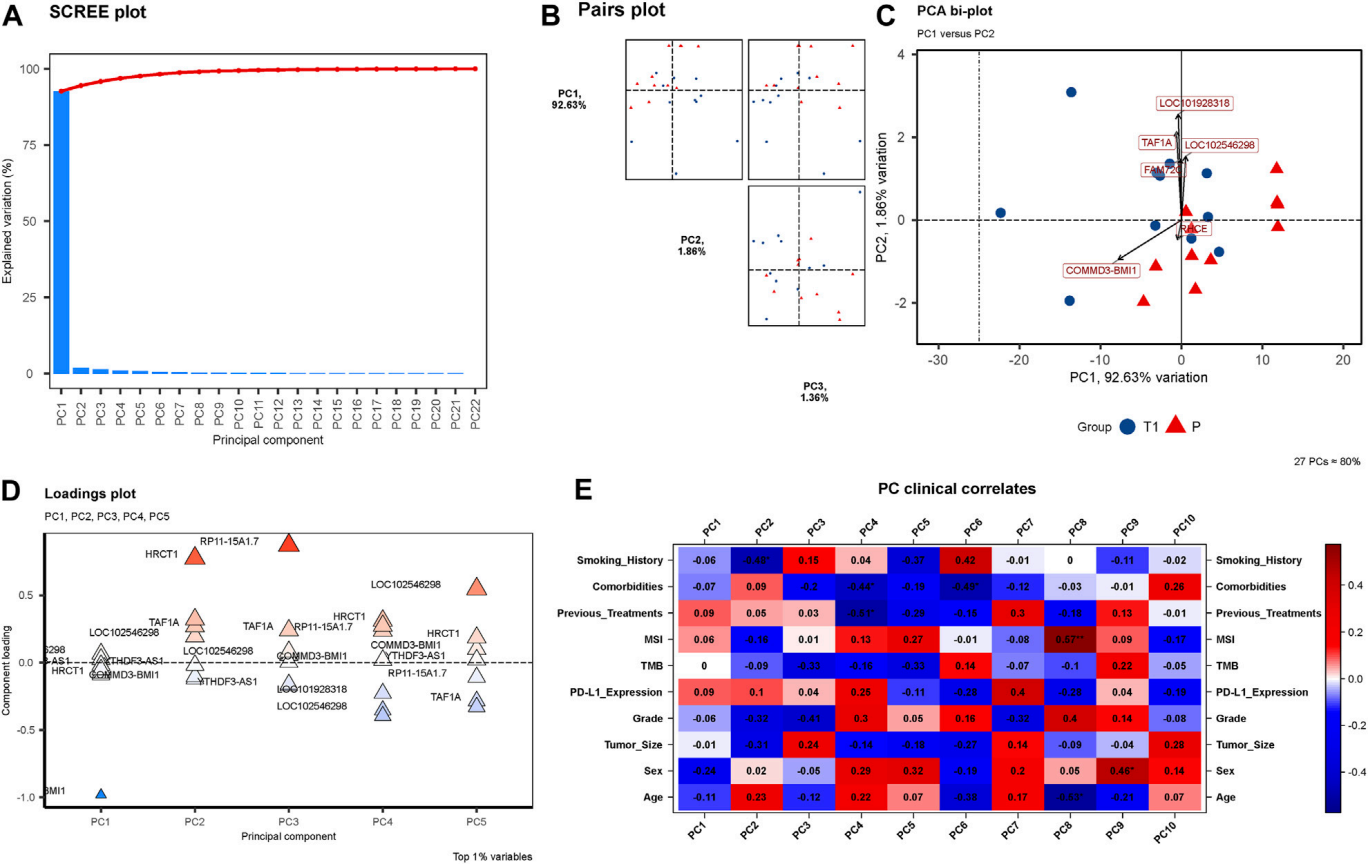
**(A)** SCREE Plot: This plot visualizes the explained variation by each of the 22 Principal Components (PCs). A precipitous decline in explained variation is evident, with a steep slope indicating that the major proportion of the variation is captured by the first few components, underscoring the dynamic changes in the transcriptomic profiles from pre-to post-treatment. **(B)** Pairs Plot: In **(B)**, a Pairs plot is showcased, highlighting the distribution of variance with PC1 accounting for a substantial 92.63%, followed by PC2 with 1.86%, and PC3 with 1.36%. The significant concentration of variance within PC1 illuminates its importance in encapsulating the transcriptomic variations observed. **(C)** PCA Bi-plot: **(C)** depicts a PCA bi-plot pinpointing six significant genes, namely COMMD3-BMI1, FAM72C, TAF1A, LOC101928318, LOC102546298, and RHCE. **(D)** Loadings Plot: **(D)** presents a Loadings plot, reaffirming the significance of the six identified genes. **(E)** PC Clinical Correlates Plot: **(E)** elucidates the correlations between ten PCs and various clinical factors. Within PC1, only Previous Treatments, MSI, and PD-L1 Expression show positive correlations, with all other factors exhibiting negative correlations.

Through rigorous PCA analysis, a discernible shift in the transcriptomic landscape of peri-tumoral hepatic tissues from pre-to post-treatment stages is observed. The SCREE plot (Figure 4A) sharply delineates a marked explained variation, predominantly encapsulated within the initial principal components, illustrating the dynamic alterations occurring in the transcriptomic profile post-treatment. In our observation from the Pairs plot (Figure 4B), a massive 92.63% of variance is encompassed by PC1, with PC2 and PC3 accounting for 1.86% and 1.36%, respectively. This substantial variance within PC1 significantly influences the overall transcriptomic landscape, underlining the pivotal role of elements contributing to PC1 in delineating the transcriptomic disparities observed. Our findings reveal six genes—COMMD3-BMI1, FAM72C, TAF1A, LOC101928318, LOC102546298, and RHCE—emerging as notably significant in Figure 4C’s PCA bi-plot (Supplementary Table S3). The Loadings plot (Figure 4D) reaffirms the significance of these six genes, consolidating their relevance and importance in understanding the intricate transcriptomic changes unfolding post-treatment. Finally, an analysis of PC Clinical Correlates (Figure 4E) reveals intriguing correlations. Within PC1, a positive correlation is noted with Previous Treatments, MSI, and PD-L1 Expression, while other factors showcase a negative correlation.

### Correlation analysis between significantly expressed genes and metabolites & animal experimental validation

**FIGURE 5:**
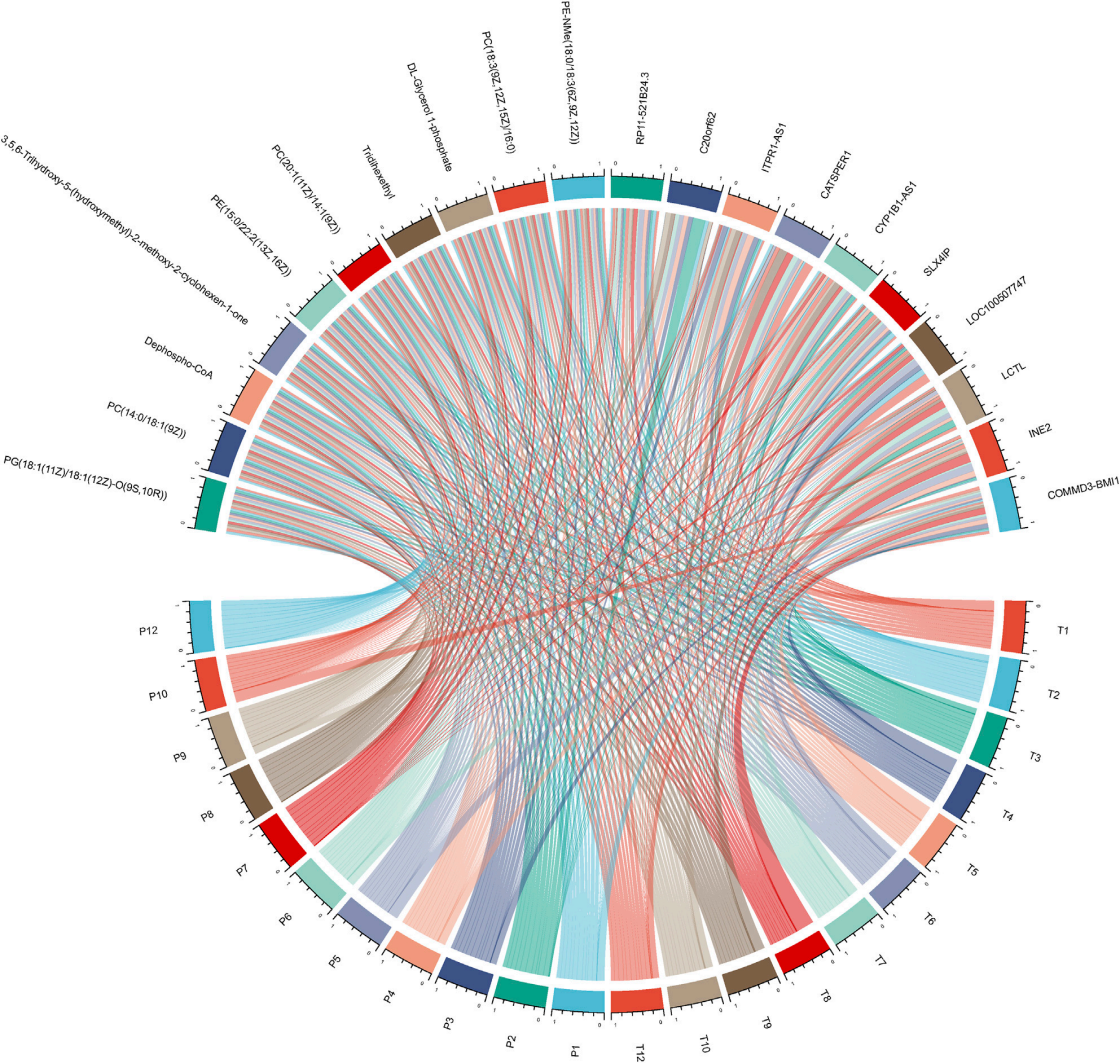
Chord Plot for Gene-Metabolite Correlation: This plot visually delineates the significant correlations between the top 10 significantly expressed genes and metabolites.

**FIGURE 6:**
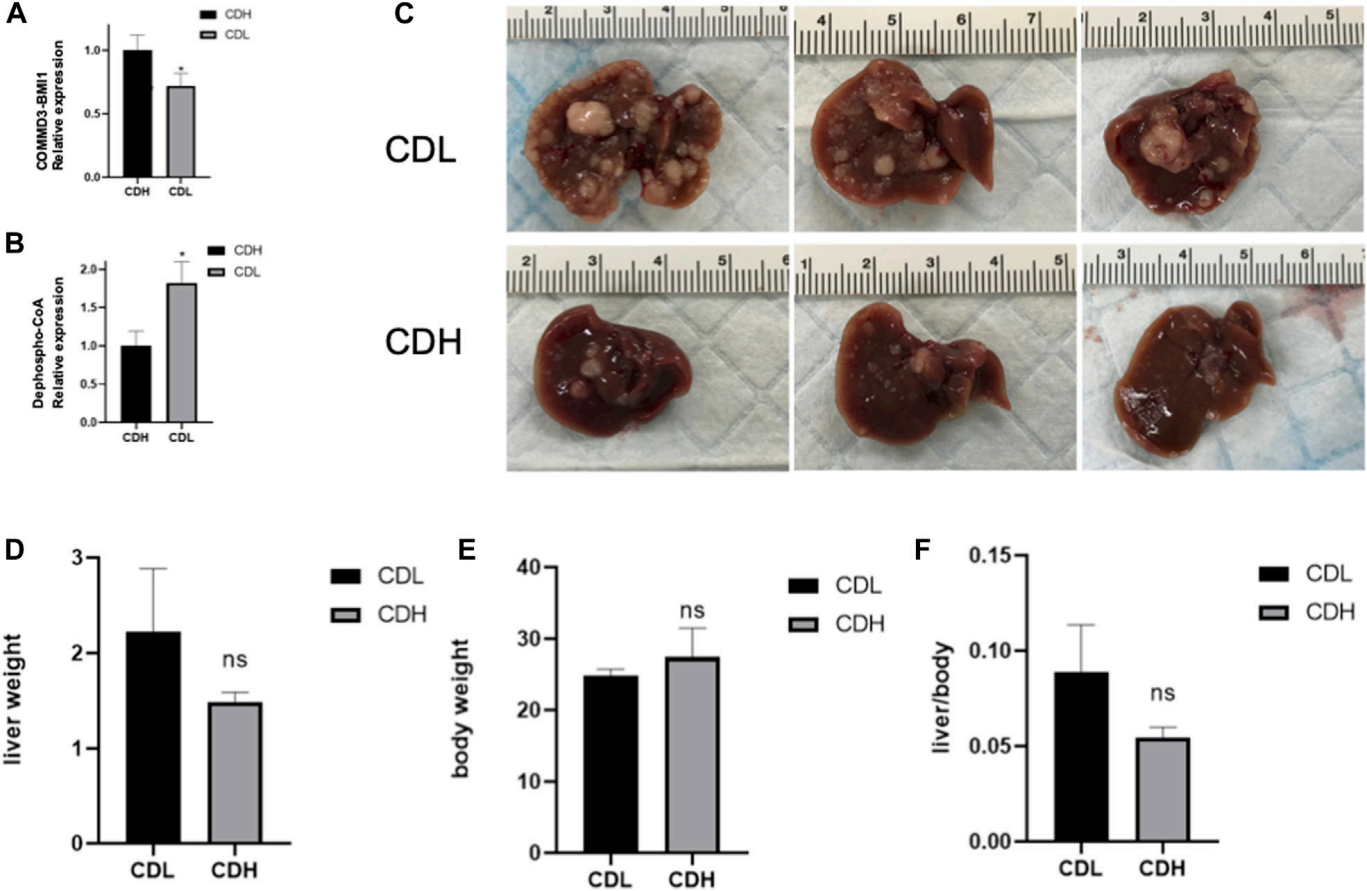
**(A)** Box Plot of COMMD3-BMI1 Expression: The Box Plot represents the distribution of COMMD3-BMI1 expression levels between the CDH and CDL groups. A significant upregulation of COMMD3-BMI1 is observed in the CDH group. *p < 0.05. **(B)** Box Plot of Dephospho-CoA Levels: The boxplot showcasing the distribution of Dephospho-CoA levels in the CDH and CDL groups. *p < 0.05. **(C)** FVB mouse livers post-immunotherapy from both CDH and CDL groups. **(D)** Liver weight of FVB mouse livers post-immunotherapy from both CDH and CDL groups. **(E)** Body weight of FVB mouse livers post-immunotherapy from both CDH and CDL groups. **(F)** Liver weight/body weight of FVB mouse livers post-immunotherapy from both CDH and CDL groups.

In an endeavor to elucidate the relationship between significantly expressed genes and metabolites, the top 10 significantly expressed genes and metabolites were selected for correlation analysis. Figure 5 showcases a chord plot that delineates the correlations uncovered during this process. A striking positive correlation was identified between COMMD3-BMI1, one of the most significantly expressed genes, and Dephospho-CoA, a prominently expressed metabolite. This compelling association hinted at potential interplay between these molecular entities in the context of hepatocellular carcinoma. To further substantiate these findings, an animal experiment was conducted. For this purpose, six FVB mice were selected and categorized into two groups: CDH and CDL. Noteworthy, the CDH group exhibited a significant overexpression of the BMI1 gene and, conversely, a marked under expression of Dephospho-CoA (Figures 6A,B). Post-immunotherapy, a discernible difference in the hepatic tumors of the subjects from each group was observed. Specifically, mice within the CDH group demonstrated heightened sensitivity to immunotherapy (Figures 6C–F).

## Discussion

Hepatocellular carcinoma (HCC), a predominant form of liver cancer, continues to present a formidable challenge to public health globally due to its intricate pathogenesis and frequently late diagnosis (Tsuchiya et al., 2015). Immune checkpoint therapies have emerged at the forefront of innovative treatments, revealing a newfound hope for patients struggling with this relentless malignancy (Leone et al., 2021). These groundbreaking therapies function by reinvigorating the immune system, thereby enabling a robust and targeted assault on tumor cells.

However, not all sunshine and roses, the therapeutic landscape of HCC is punctuated by instances of resistance to immune checkpoint therapies. This phenomenon of immunotherapy resistance is both intricate and multifaceted, often serving as a significant bottleneck to realizing the full therapeutic potential of these novel interventions (Zhang et al., 2021; Aria et al., 2022). It is within this challenging context that our study attempts to shed light on the molecular actors that might play pivotal roles in determining treatment outcomes.

In the realm of liver cancer immunotherapy, TMB and PD-L1 expression have garnered significant attention as potential predictors of therapeutic response. TMB, quantifying the number of mutations within tumor genomes, hints at the neoantigen load, which in turn can influence the ability of the immune system to recognize and combat cancer cells. A higher TMB often translates to increased neoantigens, rendering tumors more susceptible to immune checkpoint therapies. On the other hand, PD-L1 expression serves as a key immune checkpoint molecule, with its overexpression indicating an immunosuppressive tumor microenvironment, thereby providing rationale for therapies targeting the PD-L1 pathway (Li et al., 2019).

Yet, the interplay between TMB and PD-L1 expression is not straightforward. While both markers can independently predict response to immunotherapies, their combined predictive power, especially in the context of HCC, remains an active area of research. For instance, some patients with high TMB but low PD-L1 expression may still benefit from immune checkpoint therapies, while others with low TMB and high PD-L1 might not derive the expected benefit (Sholl et al., 2020). This underscores the necessity of a more nuanced understanding and perhaps a combinatorial approach to predicting treatment response. Our investigation into the tumor microenvironment and its metabolic intricacies, as detailed in the present study, adds another layer to this complex puzzle. We believe that a holistic approach, integrating insights from TMB, PD-L1 expression, and tumor microenvironmental factors, will pave the way for a more precise and effective deployment of immune checkpoint therapies in HCC.

The gene COMMD3-BMI1 has emerged as a figure of interest within our investigative lens due to its conspicuous upregulation in the pre-treatment samples (López-Nieva et al., 2019a). COMMD3-BMI1 is not merely a bystander in the cellular microcosm; it is implicated in various biological processes, including cell proliferation and survival. Its overexpression has been previously documented in different types of malignancies, suggesting its potential role as an oncogene (López-Nieva et al., 2019b; Umbreen et al., 2019). The heightened expression of COMMD3-BMI1 in our cohort might be indicative of its contributory role in fostering an environment conducive to immunotherapy resistance, warranting its further exploration as a therapeutic target or biomarker.

On the other side of the molecular spectrum resides Dephospho-CoA, a metabolite that has drawn our attention due to its significant downregulation in the CDH group. Dephospho-CoA is a crucial player in cellular metabolism, participating actively in fatty acid synthesis and energy production (Naquet et al., 2020). Its reduced levels might be reflective of altered metabolic states within the tumor microenvironment, potentially influencing the efficacy of immune checkpoint therapies (Longo et al., 2022). The downregulation of Dephospho-CoA suggests a metabolic reprogramming that might favor tumor survival and proliferation, providing a shield against the onslaught of immune cells activated by immunotherapy.

The dance between COMMD3-BMI1 and Dephospho-CoA, choreographed within the confines of hepatocellular carcinoma cells, paints a complex picture of immunotherapy resistance. This delicate molecular tango, unveiled through our study’s lens, offers tantalizing hints towards understanding the underpinnings of immunotherapy resistance in HCC. With each step and twirl, these molecules might be subtly altering the cellular stage, influencing the unfolding drama of immune-tumor interactions, and ultimately dictating the climax of therapeutic success or failure.

In conclusion, our study adds valuable brush strokes to the canvas of HCC immunotherapy, highlighting the roles of COMMD3-BMI1 and Dephospho-CoA in this intricate tableau. As we continue to decipher the molecular signatures and stories penned within tumor cells, it is imperative to acknowledge and explore the potential of these actors in steering the narrative towards a finale of therapeutic triumph over hepatocellular carcinoma. The path is long and winding, yet with each discovery, we inch closer to understanding and eventually overcoming the challenge of immunotherapy resistance in HCC.

## Data Availability

The data used in this study are publicly available in the database of the China National Center for Bioinformation (CNCB). These data can be accessed via the BioProject ID: PRJCA021673. Access to the data set can be obtained through the following link: https://ngdc.cncb.ac.cn/bioproject/browse/PRJCA021673.
